# Kinetics of Austenite Phase Transformations in Newly-Developed 0.17C-2Mn-1Si-0.2Mo Forging Steel with Ti and V Microadditions

**DOI:** 10.3390/ma14071698

**Published:** 2021-03-30

**Authors:** Mateusz Morawiec, Anna Wojtacha, Marek Opiela

**Affiliations:** Department of Engineering Materials and Biomaterials, Silesian University of Technology, 18A Konarskiego Street, 44-100 Gliwice, Poland; Anna.Wojtacha@polsl.pl (A.W.); Marek.Opiela@polsl.pl (M.O.)

**Keywords:** phase transformations, CCT diagram, dilatometry study, multi-phase steel, microstructure investigation

## Abstract

This work presents the results of phase transformation kinetics during continuous cooling in newly developed high strength low-alloy steel (HSLA). Initial theoretical calculations for the determination of heat treatment parameters were conducted. To determine the structural constituents formed due to the austenite decomposition the dilatometry approach was used. The material was cooled down from the austenitization temperature of 1000 °C with cooling rates between 0.1 °C/s to 60 °C/s. Then, light and scanning electron microscopy investigations were carried out. The microstructure after cooling at rates between 0.1 °C/s up to 1 °C/s is mainly ferritic with some fraction of granular bainite. Increasing the cooling rate led to formation of a higher fraction of bainitic ferrite. At 60 °C/s the microstructure was mainly bainite with some fraction of ferrite. To determine the presence of retained austenite, color etching using Klemm solution was used. The results show that the increase of cooling rate decreases the amount of retained austenite in the microstructure of the steel. Hardness measurements were made to determine the changes in the mechanical properties as a function of the cooling rate.

## 1. Introduction

Newly developed construction materials need to have a high strength, ductility, resistance to cracking—also at low temperatures. Sometimes, high fatigue strength is also needed. In the case of steels intended for forging, it is also very important to ensure adequate hardenability and machinability and at the same time limited cost determined by the concentration of alloy elements. Many of the listed properties require a different material development approach because their simultaneous fulfillment is a very difficult challenge for material science engineers [1,2,3].

The main problem of conventional steels intended for quenched forgings is the high concentration of carbon and alloying elements required to ensure adequate hardenability and strength as well as resistance to cracking. In addition, the large number of heat treatment and auxiliary operations significantly increases costs and production efficiency. In the last 20 years, in some applications, the manufacturing cycle of forging has been simplified, thanks to the use of thermomechanical processing technology with controlled cooling of products immediately after the completion of hot forging [4,5,6,7,8,9]. Despite many approaches, the achievement of strength at the level of quenched and tempered steels always comes at the cost of lowered impact strength and/or low fatigue strength and difficult machinability. This restriction applies to the dispersion-hardened pearlitic-ferritic steels, where the main problem is the low resistance of the pearlite to impact loads [10]. In recent years, there has been a trend of replacing pearlite with lath ferrite, bainite, and martensite. However, this does not still meet the modern needs for the use of forgings combining high strength, toughness, and fatigue strength. The main drawback of the steels produced today is the presence of carbides, which are most often the cause of stress concentration and, consequently, the initiator of impact or fatigue cracks [11].

Multiphase steels with retained austenite have the greatest potential in terms of meeting the quality requirements of forgings combining high strength, fracture toughness, and fatigue strength. The factors determining the presence of this phase lead to its stabilization thanks to the appropriate concentration of C, multi-stage heat treatment, and Si added to the steel, which contributes to a significant delay in the precipitation of cementite in bainitic ferrite [12,13,14,15,16,17,18]. Pioneering works in this area have been carried out for over twelve years, mainly in Japanese and German units [19,20,21,22,23,24,25,26,27,28,29,30]. However, the optimal parameters of hot working and cooling profiles to achieve the above-mentioned mechanical, technological, and operational properties have not been developed so far. One of the main problems is the lack of homogeneity of the austenitic phase, in particular the uncontrolled martensitic transformation of blocky grains of this phase into martensite under mechanical load conditions, which may be the cause of crack initiation and propagation during the next load cycle [20].

The necessary condition for the correct design of the conditions of thermomechanical treatment, and in particular, for the controlled multi-stage cooling of the forgings enabling the achievement of a multi-phase structure, is the knowledge of the curves of the supercooled austenite transformations. The aim of this work is the theoretical calculations of continuous-cooling-transformation (CCT) curves and experimental dilatometric tests of the newly developed 0.17C-2Mn-1Si-0.2Mo steel with Ti and V microadditions.

## 2. Material and Experiments

The chemical composition of analyzed 0.17C-2Mn-1Si-0.2Mo steel is present in Table 1. The low carbon content was selected, for good weldability. The casts of 30 mm diameter and 400 mm length were melted in a laboratory induction furnace VEM I20 type (VEM, California City, CA, USA). The designed chemical composition allows for the manufacturing of multi-phase forgings with retained austenite. The steel includes 1% Si. The Si was added to the steel because it prevents the formation of carbides during bainite formation. This leads to excess carbon, which can diffuse into the austenite increasing its thermal stability [31,32]. Because the Si has a negative impact on hot-dip galvanizing, a low content was selected. An amount of 1% was thought to be enough to inhibit the formation of carbides in bainite. At the same time 0.2% Mo, 0.031% Ti, and 0.022% V were added to the steel for strengthening and grain refinement. Ti and V form TiC, TiN, and VC carbides that during austenitization inhibit the growth of the austenite grains. This results in increased strength of the steel.

The first step was to determine the thermodynamic state using the JMatPro software (database version 12, Sente Software, Guildford, UK) general steel module [33]. A calculation of phase evolution under equilibrium conditions and continuous cooling was carried out. In this way it was possible to determine the heat treatment parameters (critical temperatures, austenitization temperature, and cooling rates to determine the continuous-cooling-transformation diagram (CCT), and to allow for the comparison of the calculations and experimental results.

To determine the real phases and verify the theoretical calculation dilatometry was used. The experiments were carried out using a BAHR dilatometer 805 A/D equipped (BÄHR-Thermoanalyse GmbH, Hüllhorst, Germany) with induction heating and a vacuum chamber. The used cooling medium was argon. Samples of 4 mm in diameter and 10 mm length were prepared for the dilatometric analysis. Determinations of the phase transformation, start and finish temperatures were carried out according to the ASTM A1033-04 standard [34].

After the dilatometric tests, the samples were prepared for metallographic investigation. The samples were cut to one third of the length and embedded in resin. Then, the specimens were ground with SiC-based papers with 220, 500, and 1200 grit. After grinding, the specimens were polished with 6, 3, and 1 µm diamond paste. In order to show the structure of the steel, etching, 5% Nital was used. For color etching after Nital, Klemm solution was used (50 mL Na_2_S_2_O_3_ in H_2_O + 1 g K_2_S_2_O_5_). Metallographic examinations were carried out using the Observer.Z1m optical microscope manufactured by Zeiss (Carl Zeiss AG, Oberkochen, Germany) and the SUPRA 25 scanning electron microscope (Carl Zeiss AG, Oberkochen, Germany). The influence of different cooling rates on the mechanical properties was investigated by hardness tests, conducted using the Vickers method with a load of 100 N.

## 3. Results and Discussion

### 3.1. Theoretical Calculations

**Figure 1:**
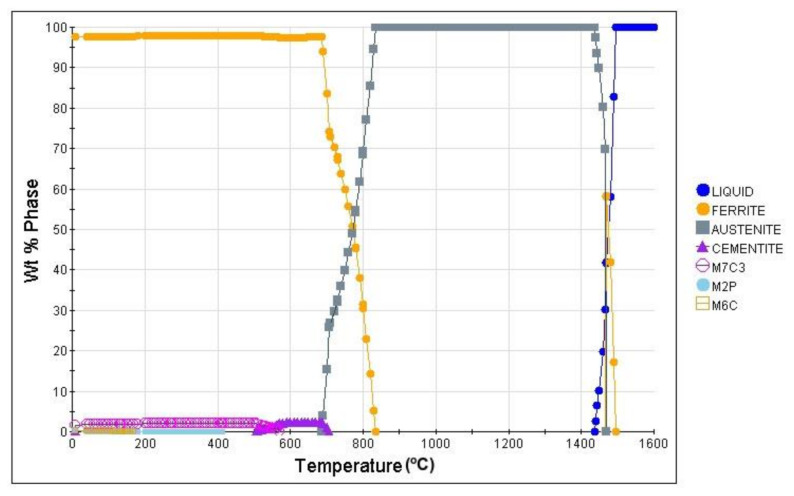
The phase evolution in steel under equilibrium conditions.

The first step of the analysis of the newly developed steel was to carry out theoretical calculations. The calculations were made with JMatPro software. The first analysis was phase evolution in steel under equilibrium conditions (Figure 1). According to this analysis, the high temperature ferrite forms at 1495 °C and is present to 1468 °C. At this temperature austenite starts to form and is present to 686 °C. In the meantime, at 836 °C ferrite starts to form. According to this analysis, the A_e1_ (austenite forming start temperature) and A_e3_ (the temperature at which the microstructure is composed of 100% austenite) temperatures are 686 and 836 °C, respectively. Cementite formation starts at 707 °C, and the highest amount of it is at 660 °C and is equal to 2.4%. Then, at 570 °C the cementite starts to dissolve, and the formation of M_7_C_3_ carbide begins. Below 200 °C, the formation of M_6_C carbide takes place.

**Figure 2:**
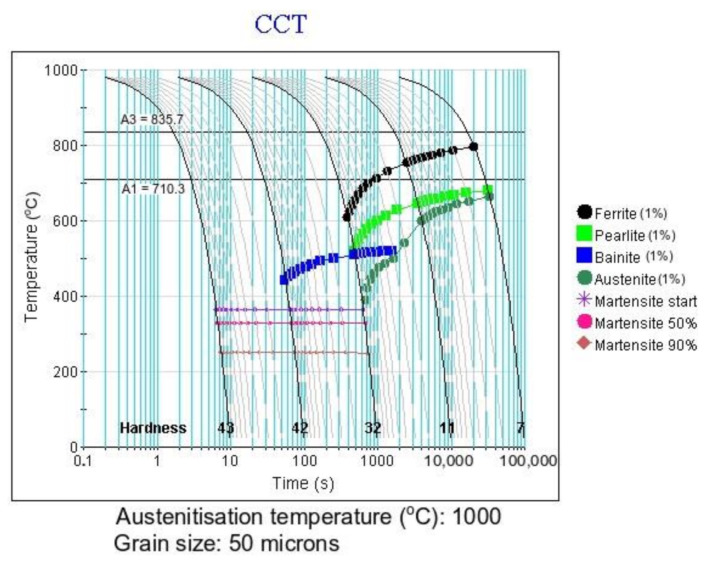
The calculated continuous-cooling-transformation (CCT) diagram of analyzed steel, together with estimated hardness (HRC).

Another calculation to determine the phase transformation kinetics during cooling at various cooling rates was the use of the continuous-cooling-transformation (CCT) diagram. The calculated diagram is presented in Figure 2. According to the result of this calculation, for cooling rates lower than 1 °C/s, a microstructure composed of ferrite and pearlite should be expected. The ferrite is present at temperatures from 797 °C to 610 °C. At the same time, pearlite is present in a temperature range of 688 °C to 532 °C, and its amount increases together with the increasing cooling rate. Additionally, at a cooling rate higher than 0.3 °C/s bainite is present in the microstructure of the steel. The starting temperature of bainite at this cooling rate is 532 °C. The amount of bainite increases with the cooling rate and after crossing the 1 °C/s it is the dominant phase. Some martensite should also be expected. For cooling rates higher than 10 °C/s, martensite should be the main phase of the steel.

### 3.2. Dilatometric Studies

**Figure 3:**
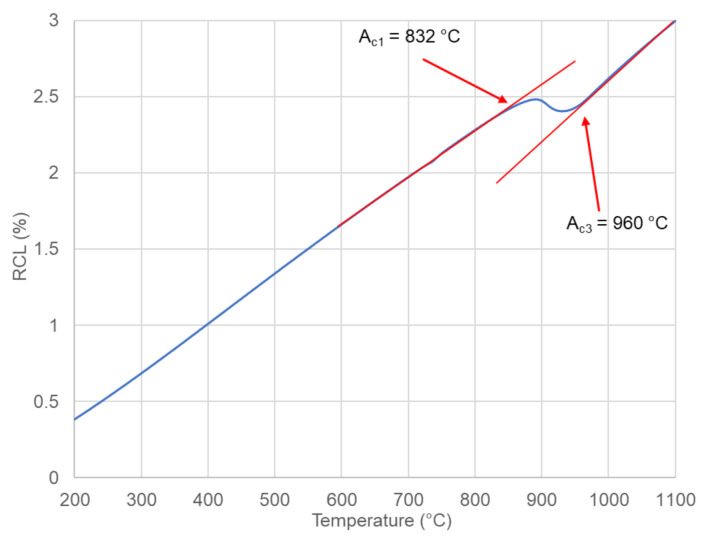
Dilatometry curve during heating for the determination of critical temperatures.

The next step of the analysis was dilatometry. It was used to determine the experimental phase transformation kinetics and to compare the calculations with the experimental results. First the A_c1_ and A_c3_ temperatures were determined at a heating rate of 3 °C/s (the heating rate used in a further dilatometry analysis). According to the results present in Figure 3 these temperatures are 832 °C and 960 °C, respectively. These temperatures were used to select the austenitization temperature of the steel. The selected austenitization temperature was 1000 °C (an A_c3_ temperature of + 40 °C to ensure a full austenitic microstructure). According to the calculation (Figure 2), to determine the CCT diagram the cooling rates from 0.1 °C/s to 60 °C/s were deemed to be sufficient. The selected cooling rates used in this experiment were: 0.1; 0.3; 0.6; 1; 2; 4; 8; 15; 30, and 60 °C/s.

**Figure 4:**
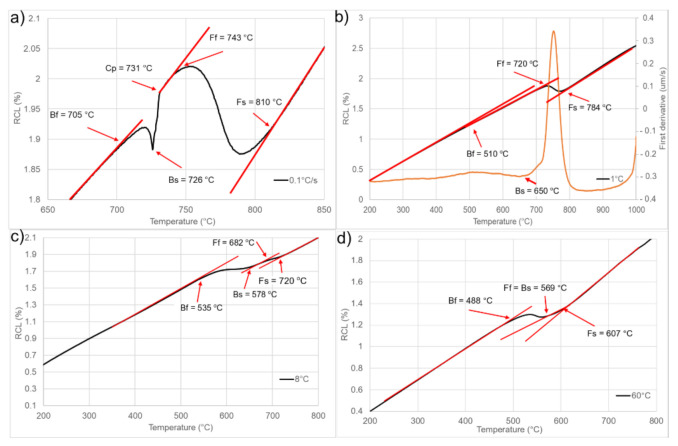
Selected dilatometric curves during cooling at different rates: (**a**) 0.1 °C/s, (**b**) 1 °C/s, (**c**) 8 °C/s, (**d**) 60 °C/s.

Knowing the austenitization temperature of the steel, heat treatments at different cooling rates were carried out. The selected results of this analysis are presented in Figure 4. The results present the dilatometric curves (black lines), which present the relative change in length (RCL) as a function of temperature. This together with the red lines, which are tangents to the dilatometric curve are the main way to determine the phase start and finish temperatures. In some cases, when the signal from the transformation is low the first derivative (orange line) can be used to determine some of the changes of the phase composition [35]. The lowest cooling rate of 0.1 °C/s (Figure 4a) shows that during cooling at 810 °C, ferrite formation starts and finishes at 743 °C. After that, the precipitation of carbides takes place. At 726 °C bainite forms. Looking at the curve, the signal from the ferrite transformation is much higher than the one from bainite. This means that the matrix will be the ferrite one with some fraction of bainite. During cooling at a rate of 1 °C/s (Figure 4b), the ferrite transformation is visible. Moreover, using the first derivative, it can be seen that bainite formation also take place. The signal is small, which is why the main phase should be ferrite. During cooling at a rate of 8 °C/s (Figure 4c) the situation is similar. During cooling, the transformations of ferrite and bainite were detected. For the highest cooling rate used in this experiment (60 °C/s—Figure 4d), the ferrite formation starts at 607 °C and finishes at 569 °C. At the same time, the bainite formation starts and is finished at 488 °C. These results mean that the analyzed steel is mostly ferritic, and very high cooling rates are necessary to form harder phases.

### 3.3. Microstructure Investigation

**Figure 5:**
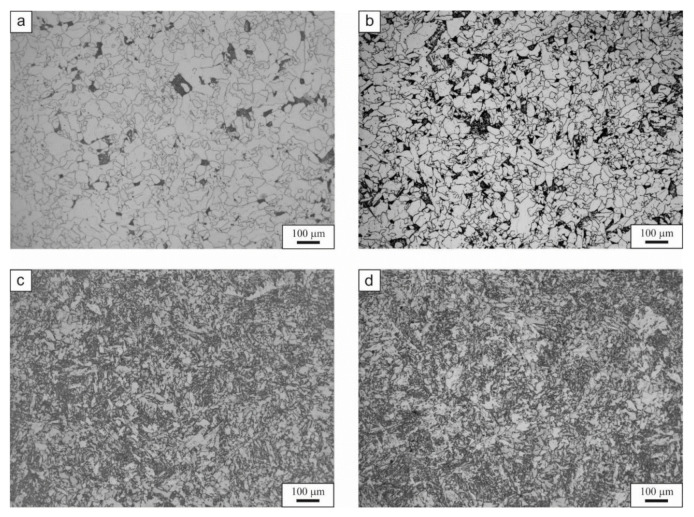
Light microscope micrographs of the selected variants of cooling: (**a**) 0.1 °C/s, (**b**) 1 °C/s, (**c**) 8 °C/s, (**d**) 60 °C/s.

**Figure 6:**
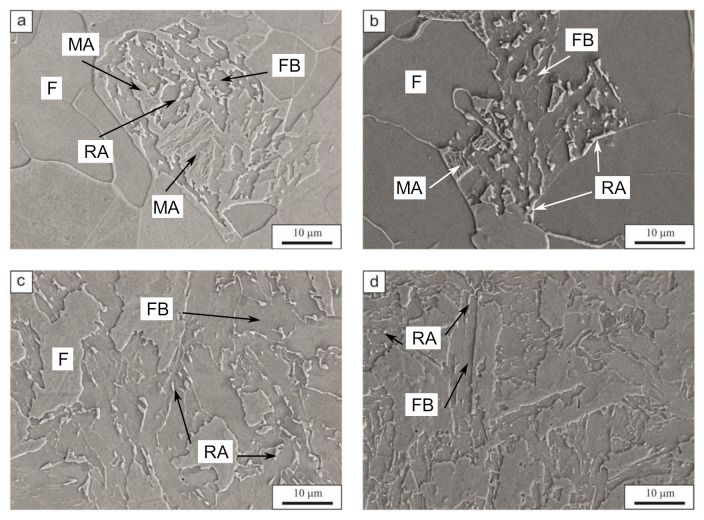
The scanning electron microscopy micrographs of the selected cooling variants: (**a**) 0.1 °C/s, (**b**) 1 °C/s, (**c**) 8 °C/s, (**d**) 60 °C/s; F—ferrite, FB—bainitic ferrite, MA—martensitic-austenitic island, RA—retained austenite.

After the dilatometric test, a microstructure investigation was carried out. The light microscope results are presented in Figure 5. According to the results, it can be seen that for cooling rates from 0.1 °C/s to 1 °C/s the main phase is ferrite with a small fraction of a dark phase. That phase is granular bainite, what can be identified at higher magnification as in Figure 6. According to Song et al. [36], the formation of granular bainite is associated with high temperature, slow cooling rates, high carbon activity gradient, and high carbon diffusion rates. This results in the appearance of carbon-poor and carbon-enriched regions. This leads during cooling to formation of larger ferrite grains between which, are carbon-rich austenite islands. These islands finally decompose into ferrite and cementite with a small fraction of retained austenite. The same conclusion was made by Qiao et al. [37] in super high strength steel. They observed that during the transformation of austenite into bainite, two different types of bainite can be formed. One is the result of formation and growth of ferrite in an equiaxed way from carbon-poor austenite regions (granular bainite). The second one is formed parallel to one other at several preferred orientations, which leads to formation of matrix by merging the ferritic laths (bainitic ferrite). In the analyzed case the first type of bainite is present in the steel. This is in agreement with dilatometry results, where the formation of ferrite matrix, carbide precipitation, and bainite formation are visible. Moreover, Zhao et al. [38] reported that the granular bainite forms at relatively high temperatures. This would explain, why the formation of granular bainite was at around 700 °C in the case of the analyzed steel. At cooling rates of 8 °C/s and higher the bainite dominates the microstructure of the steel. Additionally, the increase in cooling rate promotes the grain refinement of all phases [38]. This is because the high cooling rates decrease the available time for grains to grow.

The scanning electron microscope micrographs are presented in Figure 6. As mentioned previously, the microstructure is composed mainly of ferrite. However, at 0.1 °C/s and 1 °C/s cooling rates the presence of a local mixture of various phases between the ferrite grains is also visible. Figure 6a,b presents this mixture, which is granular bainite, together with a very small amount of martensitic–austenitic islands and retained austenite. The stabilization of retained austenite is the result of carbon enrichment during bainite formation. The high Si content prevents the formation of cementite during bainite transformation. According to Kozeschnik and Bhadeshia [39], the silicon reduces the free energy change of the transformation. This reduction slows down the kinetics of the cementite precipitation in the steel. Similar results were reported by Suzuki et al. [40], Chen et al. [41], and Caballero et al. [42]. At the higher cooling rate (1 °C/s), the granular bainite is more decomposed compared to the lower cooling rates. The 8 °C/s (Figure 6c) and 60 °C/s (Figure 6d) microstructures shows that the microstructure is composed of ferrite, bainitic ferrite, and a small fraction of retained austenite on the grain boundaries and between the bainite laths. Moreover, for the 8 °C/s variant the bainite takes a more block-like shape, which could also be described as granular bainite.

**Figure 7:**
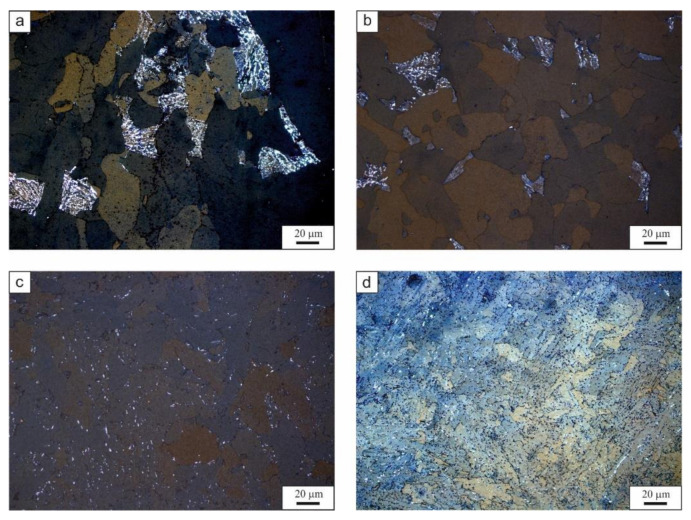
The microstructure after Klemm etching for: (**a**) 0.1 °C/s, (**b**) 1 °C/s, (**c**) 8 °C/s, (**d**) 60 °C/s. The white phase is retained austenite.

The amount of retained austenite decreases with increasing cooling rate as presented in Figure 7. This figure shows the microstructure after Klemm etching, where it is possible to distinguish the phases by color [43]. The amount of austenite (white phase) decreases at higher cooling rates. The highest amount is present in the 0.1 °C/s cooling variant. However, this phase is present only in the local zones of the granular bainite. As mentioned before, the granular bainite is surrounded by the retained austenite (Figure 7a). The amount of white phase is much smaller for the cooling rate of 1 °C/s (Figure 7b). The retained austenite is focused in the granular bainite. In case of 8 °C/s (Figure 7c), the retained austenite is visible only at the ferrite and bainite grains boundaries. The same situation arises for the 60 °C/s cooling variant. This is the result of decreasing the time available for carbon diffusion into the austenite during the formation of bainitic ferrite. The carbon needs time to diffuse into the austenite. However, increased cooling rates, decrease this time.

### 3.4. CCT Diagram

**Figure 8:**
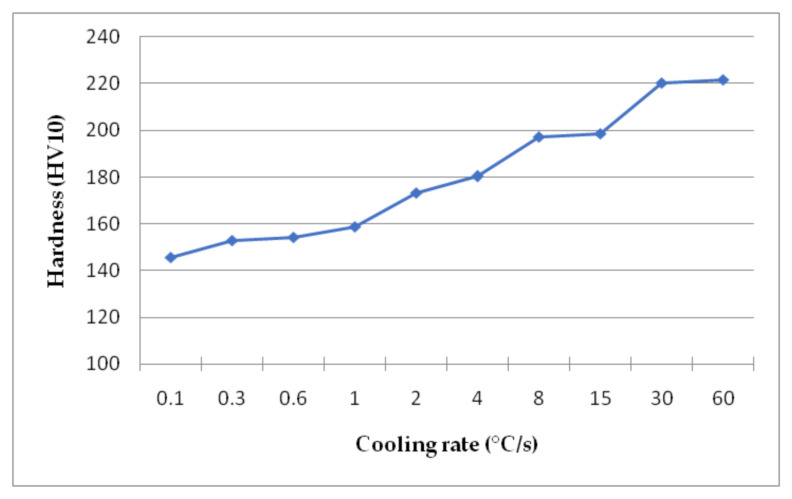
Hardness results for the samples cooled at different rates.

The last step of the research was the analysis of the hardness of the steel after cooling. The results of the hardness measurements are presented in Figure 8. The lowest hardness of the steel for the lowest cooling rate is 145 HV10. As expected, the hardness increases with increased cooling rates. This is the result of grain refinement and phase constitution changes. At the beginning the sluggish increase in hardness, is the result of grain refinement only. There is not much of change in the structural composition. Then, when the amount of bainite increases the hardness rises too. This is because the bainite as the harder phase increases the global hardness of the steel. At the same time the grain refinement of the phases has an impact upon the hardness. This is because the smaller grains increase the strength of the materials, which correlates with the Hall–Petch equation [44]. This leads to the hardness of 221 HV10 for the highest cooling rate. These results enable the formation of the CCT diagram of the analyzed steel.

**Figure 9:**
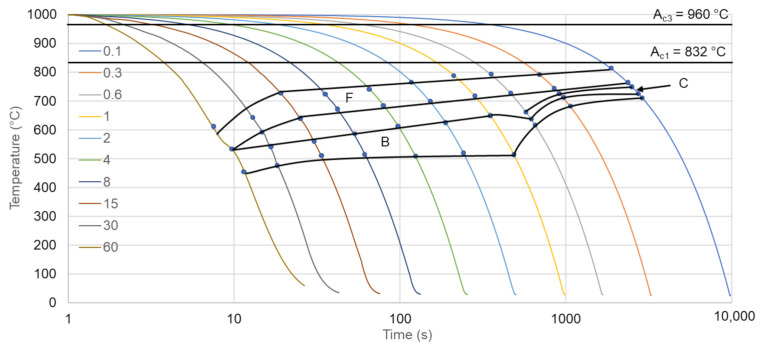
Experimental CCT diagram for analyzed steel. F—ferrite, B—bainite, C—carbides.

According to the dilatometry, microstructural, and hardness analyses a CCT diagram was created. Additionally, a comparison of the calculated and experimentally determined diagrams was carried out. The experimental CCT diagram is presented in Figure 9. According to the diagram, the steel is composed of ferrite and bainite in a range of cooling rates from 0.1 °C/s to 60 °C/s. The amount of bainite slowly increases with the cooling rate and at the same time the ferrite fraction decreases. Comparing the calculated and experimental CCT diagrams, it can be seen that the cooling rate to obtain a full martensitic structure is ca. 10 °C/s. However, from the experimental analysis the 60 °C/s rate is not enough to obtain the full martensite microstructure. All the phase areas are shifted to lower cooling rates in the case of the calculated CCT diagram. Moreover, pearlite should be expected when the cooling rate is 1 °C/s and lower. However, the results shows that there is no pearlite in the microstructure of the steel independently of the cooling rate. This is due to high Si content [39,40], which delays pearlite formation additionally, for the cooling rates higher than 1 °C/s ferrite should be absent from the microstructure. Yet, the result show that even with decreasing tendency it is still present for all cooling rates. These results mean that some level of discrepancy needs to considered when using the calculation approach, especially in newly developed multiphase steels.

## 4. Conclusions

According to the presented results of newly developed HSLA steel, it can be stated that:

- the analyzed steel does not have high hardenability. The microstructure of the steel is mainly composed of ferrite and bainite with some retained austenite, especially for lower cooling rates. To obtain a fully bainitic or martensitic microstructure, higher cooling rates are necessary.
- the presence of high Si content leads to formation of bainitic ferrite in the microstructure of the steel. The excess carbon diffuses into the austenite increasing its thermal stability. This leads to the possibility of controlling the retained austenite amount using different heat treatments.
- for the lowest cooling rate, the prior formation of ferrite results in local formation of granular bainite. The microstructure where granular bainite is formed is complex. These areas contain lath bainite and martensitic–austenitic islands.
- at cooling rates from 0.1 °C/s to 60 °C/s the hardness does not change significantly. The difference between the lowest and highest values is 76 HV10. This is the result of the grain refinement and formation of bainite.
- the comparison of the calculated and experimental CCT diagram shows some discrepancy in the results. This means that using the computational approach it is important to take into account such discrepancies during calculations, especially in the case of newly developed steels.

## Figures and Tables

**Table 1:** Chemical composition of analyzed steel (wt%).

C	Mn	P	S	Si	Mo	Cr	Ni	Ti	V
0.17	1.87	0.014	0.020	1.0	0.22	0.028	0.018	0.031	0.022

## Data Availability

Data sharing is not applicable to this article.
